# Lung Cancer Susceptibility and *hOGG1* Ser326Cys Polymorphism: A Meta-Analysis

**DOI:** 10.3390/cancers2041813

**Published:** 2010-10-28

**Authors:** Chikako Kiyohara, Koichi Takayama, Yoichi Nakanishi

**Affiliations:** 1 Department of Preventive Medicine, Graduate School of Medical Sciences, Kyushu University, 3-1-1 Maidashi, Higashi-ku, Fukuoka 812-8582, Japan; 2 Research Institute for Diseases of the Chest, Graduate School of Medical Sciences, Kyushu University, 3-1-1 Maidashi, Higashi-ku, Fukuoka 812-8582, Japan; E-Mails: koichi-t@kokyu.med.kyushu-u.ac.jp (K.T.); yoichi@kokyu.med.kyushu-u.ac.jp (Y. N.)

**Keywords:** epidemiology, genetic polymorphism, lung cancer, meta-analysis, human 8-oxoguanine DNA glycosylase 1 (hOGG1)

## Abstract

Recent lung cancer studies have focused on identifying the effects of single nucleotide polymorphisms (SNPs) in candidate genes, among which DNA repair genes are increasingly being studied. Genetic variations in DNA repair genes are thought to modulate DNA repair capacity and are suggested to be related to lung cancer risk. In this study, we tried to assess reported studies of association between polymorphism of human 8-oxoguanine DNA glycosylase 1 (*hOGG1*) Ser326Cys and lung cancer. We conducted MEDLINE, Current Contents and Web of Science searches using "hOGG1", "lung cancer" and "polymorphism" as keywords to search for papers published (from January 1995 through August 2010). Data were combined using both a fixed effects (the inverse variance-weighted method) and a random effects (DerSimonian and Laird method) models. The Cochran Q test was used for the assessment of heterogeneity. Publication bias was assessed by both Begg’s and Egger’s tests. We identified 20 case-control studies in 21 different ethnic populations. As two studies were not in the Hardy-Weinberg equilibrium, 18 case-control studies in 19 different ethnic populations (7,792 cases and 9,358 controls) were included in our meta-analysis. Summary frequencies of the Cys allele among Caucasians and Asians based on the random effects model were 20.9% (95% confidence interval (CI) = 18.9–22.9) and 46.1% (95% CI = 40.2–52.0), respectively. The distribution of the Cys allele was significantly different between Asians and Caucasians (P < 0.001). The Cys/Cys genotype was significantly associated with lung cancer risk in Asian populations (odds ratio = 1.27, 95% CI = 1.09–1.48) but not in Caucasian populations. This ethnic difference in lung cancer risk may be due to environmental factors such as cigarette smoking and dietary factors. Although the summary risk for developing lung cancer may not be large, lung cancer is such a common malignancy that even a small increase in risk can translate to a large number of excess lung cancer cases. As lung cancer is a multifactorial disease, further investigations of the gene-gene and gene-environment interactions on the *hOGG1* polymorphism-associated lung cancer risk may help to better understand of the molecular pathogenesis of human lung cancer.

## 1. Introduction

Sporadic cancer is a multifactorial disease that results from complex interactions between many genetic and environmental factors [1]. This means that there will not be a single gene or single environmental factor that has large effects on cancer susceptibility. Environmental factors (e.g., tobacco smoke, dietary factors, infectious agents and radiation) add to the carcinogenic load to which humans are exposed, but exact numbers for added risk are generally less well established.

Cigarette smoke contains several thousand chemicals that are known to chemically modify DNA [2] and lead to the formation of mutations [3]. Most of these compounds are procarcinogens that must be activated by Phase I enzymes, such as cytochrome P450s. All activated carcinogens such as benzo(a)pyrene 7,8-diol 9,10-epoxide (BPDE) and *N*-hydroxy-4-aminobiphenyl can bind to DNA and form DNA adducts that are capable of inducing mutations and initiating carcinogenesis. The capacity to repair DNA damage induced by activated carcinogens appears to be one of the host factors that may influence lung cancer risk. A critical cellular response that counteracts the carcinogenic effects of DNA damage is DNA repair. On the basis of recent investigations, various DNA repair mechanisms function continuously to correct damaged DNA that is caused by exposure to either endogenous factors, such as estrogen, or exogenous toxic substances, such as cigarette smoking, dietary factors, *etc*.

Several studies have investigated whether reduced DNA repair capacity (DRC) is associated with an increased risk of cancer [4]. The reduced DRC of BPDE-DNA adducts is associated with an increased risk of lung cancer (2.1-fold, 95% confidence interval (CI) = 1.5–3.0) [5]. The reduced DRC has been shown to be associated with a 5.7-fold (95% CI = 2.1–15.7) increased risk of developing lung cancer [6]. Likewise, the reduced DRC of bleomycin-induced damage was found to be associated with an increased risk of lung cancer [7]. These studies suggested that a low DRC of various DNA repair mechanisms predisposes individuals to lung cancer and this realization prompted us to search for defined DNA repair activities that may be risk factors for lung cancer. Polymorphisms in DNA repair genes may be associated with differences in the DRC of DNA damage and may influence an individual’s risk of lung cancer, because the variant genotype in those polymorphisms might destroy or alter repair function.

It is believed that the predominant pathway used for removal of oxidized and many of the alkylated bases is base excision repair (BER). The process of BER is initiated by DNA glycosylases (e.g., human 8-oxoguanine DNA glycosylase 1 (hOGG1), endonuclease III homolog 1, thymine glycol-DNA-glycosylase), which are often promiscuous as far as their substrate specificity is concerned. The glycosylase hydrolyzes the *N*-glycosylic bond between the oxidized base and sugar moiety, thus releasing the free damaged base and giving rise to an apurinic/apyrimidinic (AP) site. AP endonuclease (APE) acts upon the AP site generating a single strand break by cleaving the phosphodiester backbone 5′ to the AP site, leaving behind a 3'-hydroxyl group and a 5'-deoxyribose phosphate group. At this point the BER pathway can proceed through two different sub-pathways: short-patch and long-patch BER. These pathways are differentiated by the enzymes involved and the number of nucleotides removed. Short-patch BER replaces a single nucleotide by polymerase β and the newly synthesized DNA is sealed by the DNA ligase III/X-ray cross-complementing group 1 heterodimer [8]. Long-patch BER inserts 2–13 nucleotides by the concordant action of polymerase δ, proliferating cell nuclear antigen, flap endonuclease 1 and ligase I.

Oxidative stress induces a cellular redox imbalance which has been found to be present in various cancer cells compared with normal cells; the redox imbalance thus may be related to oncogenic stimulation. DNA mutation is a critical step in carcinogenesis and elevated levels of oxidative DNA lesions have been noted in various tumors, strongly implicating such damage in the etiology of cancer. It appears that the DNA damage is predominantly linked with the initiation process. By far the most studied oxidative DNA lesions are 8-hydroxyguanine (8-oxoG) and its 2'-deoxynucleoside equivalent, 7,8-dihydro-8-oxo-2'-deoxyguanosine. It appears that, quantitatively, BER is the most important route for the removal of the majority of oxidative lesions. The glycosylase considered to have the primary responsibility for the removal of 8-oxoG in human cells is the hOGG1 [9]. The activity of hOGG1 is complemented by another enzyme, denoted hOGG2, which removes the 8-oxoG from the nascent strand in 8-oxoG:A or 8-oxoG:G pairs, arising from misincorporation of 8-OH-dGTP [10].

Among DNA repair genes that encode DNA repair proteins, the hOGG1 DNA repair enzyme and its association with lung cancer risk may deserve special attention. The *hOGG1* maps on chromosome 3, at 3p26.2. At least 20 validated sequence variants have been described to date. Among those, a C/G sequence variant leading to an amino acid change from serine to cysteine at codon 326 (Ser326Cys, rs1052133) has been studied most frequently. Several *in vivo* or *in vitro* studies have examined the association between *hOGG1* genotypes and enzyme activity, though the results have been inconsistent, as reviewed by Weiss *et al.* [11]. The Ser326Cys polymorphism has not yet been convincingly shown to cause decreased hOGG1 activity in humans, although a recent study found higher levels of oxidized guanine in human lymphocyte DNA from subjects with the Cys/Cys genotype as compared with subjects with Ser/Ser genotype or Ser/Cys genotype after *ex vivo* treatment with sodium dichromate [12]. Sugimura *et al.* first demonstrated that the *hOGG1* Ser326Cys polymorphism was not associated with lung cancer risk [13]. Subsequent studies in different populations produced mixed results. Several studies [14,15,16,17,18,19,20,21,22,23,24,25,26,27] have replicated the finding from the first study, but other studies [28,29,30,31,32] reported that the *hOGG1* Ser326Cys polymorphism is associated with lung cancer risk. Given the amount of accumulated data and the still equivocal role of the *hOGG1* Ser326Cys polymorphism in the etiology of lung cancer in general, we decided to perform a meta-analysis of all published studies on the association between the *hOGG1* Ser326Cys polymorphism and lung cancer. Since a more precise estimation of the association helps us better understand the possible risk of lung cancer, in this study, we have included several additional epidemiologic studies which allowed for a greater number of subjects, and excluded several ineligible studies which allowed for a more accurate risk estimation than in prior meta-analysis [33].

## 2. Materials and Methods

### 2.1. Identification and Eligibility of Relevant Studies

We conducted MEDLINE, Current Contents and Web of Science searches using "hOGG1", "lung cancer" and "polymorphism" as keywords to search for papers published (from January 1995 through August 2010). Additional articles were identified through the references cited in the first series of articles selected. Articles included in the meta-analysis were in English language, using human subjects, published in the primary literature and had no obvious overlap of subjects with other studies. We excluded studies with the same data or overlapping data by the same authors. Case-control studies were eligible if they had determined the distribution of the relevant genotypes in lung cancer cases and in concurrent controls using a molecular method for genotyping. Using the MEDLINE database, we identified 20 genetic epidemiological studies that provided information on lung cancer occurrence associated with the *hOGG1* Ser326Cys polymorphism. No additional articles through Current Contents or Web of Science have been identified.

### 2.2. Data Extraction and Assessment of Study Quality

For each study, characteristics such as authors, year of publication, ethnic group of the study population, source of control population, number of genotyped cases and controls, crude odds ratio (OR) and the method for quality control of genotyping were noted. For studies including subjects of different ethnic groups, data were extracted separately for each ethnic group whenever possible.

Methods for defining study quality are more clearly delineated in genetic studies than those for observational studies. We combined only studies with allelic frequencies being in Hardy-Weinberg equilibrium (HWE) (Pearson χ^2^ test, P ≥ 0.05) because departure from HWE can imply the presence of genotyping error, possible ethnic admixture in the population or selection bias (lack of representativeness of the general population). We assessed the homogeneity of the study population (Caucasian or Asian).

### 2.3. Meta-Analysis

Data were combined using both a fixed effects (the inverse variance-weighted method) and a random effects (DerSimonian and Laird method) models [34]. The Cochran Q test is used for the assessment of heterogeneity. The fixed effects model is used when the effects are assumed to be homogenous, while the random effects model is used when they are heterogenous. In the absence of between-study heterogeneity, the two methods provide identical results. The presence of heterogeneity can result from differences in the selection of controls, age distribution, prevalence of lifestyle factors, histological type of lung cancer, stage of lung cancer, *etc*. The random effects model incorporates an estimate of the between-study variance and tends to provide wider CIs when the results of the constituent studies differ among themselves. As the random effects model is more appropriate when heterogeneity is present [34], the summary OR and prevalence were essentially based on the random effects model. The meta-analyses were performed on crude ORs, since the adjusted ORs were not comparable because of the inclusion of different covariates in the multivariate regression models. Using individuals with the homozygous common genotype as the reference group, we calculated ORs for individuals with the heterozygous genotype and homozygous rare genotype separately. The Q statistic was considered significant for P < 0.10 [35,36]. Publication bias is always a concern in meta-analysis. The presence of publication bias indicates that non-significant or negative findings remain unpublished. To test for publication bias, both Begg’s (regression method) [37] and Egger’s (rank correlation approach) [38] tests are commonly used to assess whether smaller studies reported greater associations than larger studies. Furthermore, the newly developed Trim and Fill method was also applied to test the presence of publication bias [39]. Publication bias is considered significant for P < 0.10. For each genetic comparison, subgroup analysis was stratified by the ethnicity or the source of controls and, if possible, histological type of lung cancer. All of the calculations were performed using STATA Version 10.1 (Stata Corporation, College Station, TX) software.

## 3. Results

### 3.1. Description of Individual Studies

We identified 20 case-control studies in 21 different ethnic populations. Table 1 shows the individual ORs from each study and summary ORs of the *hOGG1* Ser326Cys polymorphism [13,14,15,16,17,18,19,20,21,22,23,24,25,26,27,28,29,30,31,32] In two studies [19,21], genotype distributions in control population deviated from the HWE. The first study among Japanese indicated the Ser allele frequency was similar between controls (0.59) and lung cancer cases (0.59) and OR for the Cys/Cys genotype was 1.13 (95% CI = 0.63–2.02) [13]. Six Asian studies also found no association between the *hOGG1* Ser326Cys polymorphism and lung cancer risk [15,16,17,19,23,26]. However, one of them found that OR for carrying at least one copy of the risk allele significantly increased in a dose-dependent manner with allele number among Japanese [23]. Two Asian studies found that the Cys/Cys genotype was marginally associated with an increased risk of lung cancer [31,32]. Significantly increased risk for lung cancer was observed for the Cys/Cys genotype (OR = 4.10, 95% CI = 1.65–10.2) among mostly composed of Caucasians [29]. Six Caucasian (mostly composed of Caucasians) studies reported that the Cys allele was not significantly associated with an increased risk of lung cancer [14,18,20,21,22,24]. A significantly decreased risk for lung cancer in individuals heterozygous for the Ser326Cys polymorphism was observed among Caucasians (adjusted OR = 0.51, 95% CI = 0.27–0.95) [30]. Compared with the Ser/Ser genotype, the Cys/Cys genotype was associated with an increased risk of lung cancer (OR = 1.76, 95% CI = 1.15–2.71) among a mixed population [28]. In Turkish, Latino and African-American populations there was not a significant association between lung cancer risk and the Ser326Cys polymorphism [25,27]. In a narrative review, the *hOGG1* Ser326Cys polymorphism has inconsistently been associated with risk of lung cancer as recently reviewed with ORs above unity for subjects with two mutant alleles in five studies, of which two were statistically significant and two studies like the present showing an OR below unity [11].

**Table 1:** Description of the studies included in the meta-analyses of the association between the *hOGG1* Ser326Cys polymorphism and lung cancer.

Author, published year [reference no.]	Ethnicity	No. of Cases/ Controls	Source of controls	OR (95% CI)*		Prevalence of the Cys allele in controls	Hardy-Weinberg test, P	Quality control of genotyping
				Ser/Cys	Cys/Cys			
Sugimura *et al.*, 1999 [13]	Asian	241/197	Hospital	0.80 (0.52–1.21)	1.13 (0.63–2.02)	0.409	0.08	Sequencing
Wirkman *et al.*, 2000 [14]	Caucasian	105/105	Hospital	0.66 (0.37–1.17)	2.20 (0.41–11.8)	0.224	0.07	Sequencing
Ito *et al.*, 2002 [15]	Asian	138/240	Hospital	1.02 (0.63–1.67)	0.85 (0.46–1.56)	0.471	0.84	None
Sunaga *et al.*, 2002 [16]	Asian	198/152	Hospital	1.49 (0.91–2.43)	0.98 (0.54–1.77)	0.454	0.13	None
Le Marchand *et al.*, 2002 [28]	Admixture	298/405	Population	0.90 (0.65–1.26)	1.76 (1.15–2.71)	0.347	0.35	Sequencing
Lan *et al.*, 2004 [17]	Asian	118/109	Population	1.96 (1.10–3.48)	1.84 (0.83–4.06)	0.335	0.23	None
Park *et al.*, 2004 [29]	Caucasian	179/350	Population	1.89 (1.27–2.80)	4.10 (1.65–10.2)	0.147	0.86	Sequencing
Vogel *et al.*, 2004 [18]	Caucasian	256/269	Population	1.09 (0.75–1.60)	0.78 (0.35–1.72)	0.240	0.24	Replication**
Liang *et al.*, 2005 [19] ‡	Asian	227/227	Hospital	0.94 (0.63–1.41)	0.98 (0.33–2.87)	0.606	0.04	Sequencing
Hung *et al.*, 2005 [20]	Caucasian	2,155/2,163	Hospital	0.90 (0.79–1.03)	1.15 (0.84–1.57)	0.202	0.22	Replication**
Zienolddiny *et al.*, 2006 [21] ‡	Caucasian	326/386	Population	0.91 (0.64–1.29)	0.63 (0.40–0.97)	0.346	0.00015	Replication†
Matullo *et al.*, 2006 [22]	Caucasian	116/1,094	Population	1.26 (0.83–1.91)	0.82 (0.21–2.33)	0.215	0.90	Replication**
Kohno *et al.*, 2006 [23]	Asian	1097/394	Hospital	1.24 (0.94–1.63)	1.43 (1.02–2.01)	0.447	0.63	None
Sorensen *et al.*, 2006 [24]	Caucasian	431/796	Population	1.04 (0.80–1.35)	1.18 (0.63–2.21)	0.220	0.25	Replication**
De Ruyck *et al.*, 2007 [30]	Caucasian	110/110	Hospital	0.58 (0.33–1.02)	0.61 (0.13–2.82)	0.245	0.18	None
Karahlil *et al.*, 2008 [25]	Turkish	165/250	Hospital	0.82 (0.54–1.24)	0.65 (0.32–1.29)	0.328	0.55	None
Miyaishi *et al.*, 2009 [26]	Asian	208/121	Hospital	1.47 (0.79–2.73)	1.34 (0.65–2.77)	0.455	0.27	None
Chang *et al.*, 2009 [27]	Latino	112/296	Population	0.91 (0.56–1.47)	1.05 (0.45–2.32)	0.321	0.52	Replication**
Chang *et al.*, 2009 [27]	African-American	254/280	Population	1.32 (0.89 –1.98)	0.89 (0.25–3.00)	0.154	0.69	Replication**
Chang *et al.*, 2009 [31]	Asian	1,096/997	Population	1.17 (0.89–1.52)	1.31 (0.99–1.73)	0.604	0.74	Replication **
Okasaka *et al.*, 2009 [32]	Asian	515/1,030	Hospital	1.00 (0.77–1.33)	1.27 (0.93–1.75)	0.493	0.08	None

* Crude odds ratio and 95% confidence interval. ** Random samples †All samples; ‡ Excluded from the meta-analysis because genotype distribution of control population was not in Hardy-Weinberg equilibrium.

### 3.2. Quantitative Synthesis

**Figure 1:**
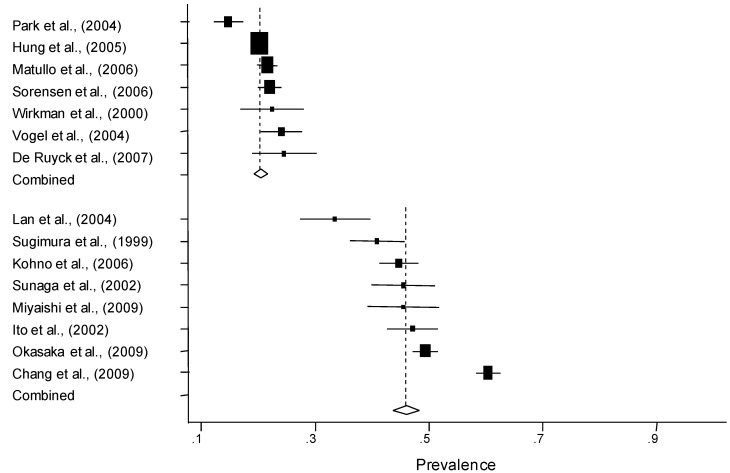
The Cys allele frequency of seven Caucasian populations and eight Asian populations among controls. The center of a box and the horizontal line indicate the prevalence and the 95% CI in each study, with the areas of the box representing the weight of each study. The summary prevalence based on the random effects model is represented by the middle of a diamond whose width indicates the 95% CI. The summary prevalence is also shown by the dotted vertical line. The summary prevalence of Caucasians and Asians based on the random effects model are 20.9% (95% CI = 18.9–22.9; Cochran Q test: Q statistic = 28.23, P < 0.0001) and 46.1% (95% CI = 40.2–52.0; Cochran Q test: Q statistic = 144.7, P < 0.0001), respectively. The distribution of the Cys allele was significantly different between Asians and Caucasians (P < 0.001).

As two studies were not in the HWE, 18 case-control studies in 19 different ethnic populations (7,792 cases and 9,358 controls) were included in our meta-analysis. In stratified analysis by ethnicity (Caucasian or Asian), 15 populations were included in the analysis. As shown in Table 2, summary frequencies of the Cys allele among Caucasian and Asian controls based on the random effects model were 20.9% (95% CI = 18.9–22.9) and 46.1% (95% CI = 40.2–52.0), respectively. Studies included in the meta-analysis in ascending order of the Cys allele frequency by ethnic group are presented in Figure 1. As shown in Figure 1 and Table 2, the distribution of the Cys allele among controls was significantly different between Asians and Caucasians (P < 0.001). As for Caucasian controls, the prevalence of the Cys allele among hospital-based case-control studies (21.1%, 95% CI = 18.8–23.3%) was slightly higher than that among population-based case-control studies (20.5%, 95% CI = 17.1–23.9%). Concerning Asian controls, the prevalence of the Cys allele among population-based and hospital-based case-control studies were 47.1% (95% CI = 20.7–73.5%) and 45.8% (95% CI = 43.1–48.5%), respectively. The former estimate based on only two studies was associated with a wide CI for the prevalence of the Cys allele.

**Table 2:** The summary prevalence of the Cys allele in controls.

Subgroup		No. of populations	Number of controls	Prevalence of the Cys allele (%)*
Ethnicity				
Caucasian		7	4,887	20.9 (18.9–22.9)
Asian		8	3,240	46.1 (40.2–52.0)
Design				
Population-based	Overall	9	4,596	28.7 (17.9–39.4)
	Caucasian	4	2,509	20.5 (17.1–23.9)
	Asian	2	2,212	47.1 (20.7–73.5)
Hospital-based	Overall	10	4,762	37.3 (27.6–47.0)
	Caucasian	3	2,178	21.1 (18.8–23.3)
	Asian	6	2,134	45.8 (43.1–48.5)

* Based on random effects model

**Figure 2:**
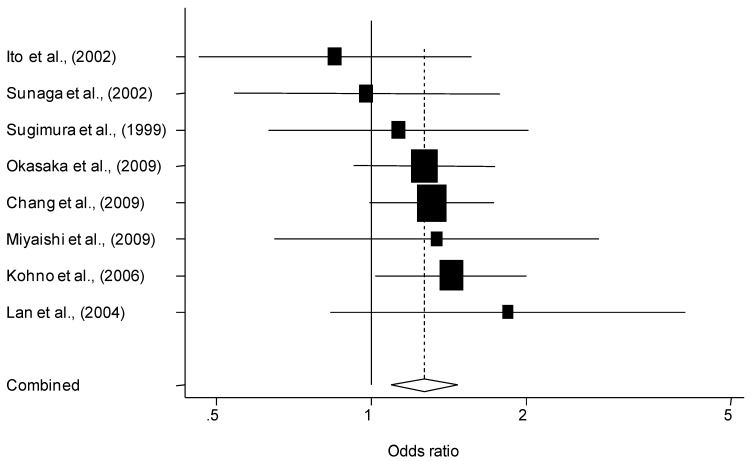
Meta-analysis of eight Asian studies of lung cancer and the *hOGG1* Ser326Cyspolymorphism (Cys/Cys *vs.* Ser/Ser). The center of a box and the horizontal line (logarithm) indicate the odds ratio (OR) and the 95% confidence interval (CI) in each study, with the areas of the boxes representing the weight of each study. The summary OR based on random effects model is represented by the middle of a diamond whose width indicates the 95% CI. The summary OR is shown by the dotted vertical line. Summary OR was 1.27 (95% CI = 1.09–1.48; Cochran Q test: Q statistic =3.93, P = 0.79).

As shown in Table 3, combining data from all 19 populations on the basis of 7,792 cases and 9,358 controls, the summary ORs were 1.07 (95% CI = 0.95–1.21) for Ser/Cys carriers and 1.24 (95% CI = 1.09–1.42) for Cys/Cys carriers. There was a significant association between lung cancer risk and the *hOGG1* Ser326Cys polymorphism among Asians but not in Caucasians. The summary ORs for the Cys/Cys genotype among Caucasians (mostly composed of Caucasians) and Asians were 1.24 (95% CI = 0.84–1.83) and 1.27 (95% CI = 1.09–1.48), respectively. Asian individual studies included in the meta-analysis in ascending order of the OR for the Cys/Cys genotype are shown in Figure 2. In the case of the summary OR for Ser/Cys genotype, heterogeneity was present in the analyses of all studies combined and Caucasian studies combined. In the stratified analysis by both study design and ethnicity, a significant increased risk was found both in population-based (OR = 1.36, 95% CI = 1.05–1.77) and hospital-based (OR = 1.23, 95% CI = 1.02–1.48) case-control studies among Asians. Unlike Caucasian control populations, who were recruited through either population-based methods or hospitals, 75% of Asian control populations were recruited by using hospital-based methods. Among Caucasian populations, there were no differences in estimates based on whether the control source was healthy or hospital based. A further analysis on histological type was performed to assess whether the impact of the *hOGG1* Ser326Cys polymorphism between adenocarcinoma, squamous cell carcinoma and small cell carcinoma cases (the three histological types present most often in the data set) was similar or not. Among the eight case-control studies (2,707 lung cancer cases and 4,479 controls), the summary OR for the Cys/Cys genotype in adenocarcinoma was 1.43 (95% CI = 1.19-1.72). Among both Caucasians (612 cases and 2,618 controls) [14,20,29] and Asians (2,095 cases and 1,861 controls) [13,16,23,26,31], subjects with the Cys/Cys genotype were at increased risk of adenocarcinoma. Summary ORs for Caucasians and Asians were 1.90 (95% CI = 0.99–3.63, P = 0.054) and 1.38 (95% CI = 1.13–1.69), respectively. Meanwhile, it was found that increased risk associated with the Cys/Cys genotype was not evident for squamous cell lung cancer risk among both Caucasians and Asians. Summary ORs for Caucasians and Asians were 1.56 (95% CI = 0.54–4.48) [14,20,29] and 1.07 (95% CI = 0.74–1.53) [13,26,31], respectively. The available data on small cell carcinoma were insufficient [13,31]. Evidence for heterogeneity was absent in the analyses in subjects with the Cys/Cys genotype The Begg’s and Egger’s tests for publication bias were not statistically significant in all analyses except for the Cys/Cys genotype with squamous cell carcinoma in the case of all populations combined. Although the regression method is more sensitive than the rank correlation approach, both the Begg’s (P = 0.091) and Egger’s (P = 0.097) tests were statistically significant for publication bias. Therefore, the potential presence of publication bias was further evaluated using the Trim and Fill analysis. Trimming was based on the fixed-effects model and the summary OR obtained by using a random-effects model was 0.96 (95% CI = 0.67–1.36). Thus, the result that there is no association between lung cancer and the Cys/Cys genotype with squamous cell carcinoma in the case of all populations combined remained unchanged.

**Table 3:** Summary of OR for the *hOGG*1 Ser326Cys polymorphism and lung cancer.

Subgroup		No. of populations	No. of cases / controls	Ser/Cys vs. Ser/Ser	Cys/Cys *vs.* Ser/Ser	Cochran Q test for heterogeneity, P
						Ser/Cys	Cys/Cys
Ethnicity							
Caucasian		7	3,352/4,887	1.02 (0.81–1.29)	1.24 (0.84–1.83)	0.004	0.13
Asian		8	3,611/3,240	1.16 (1.00–1.36)*	1.27 (1.09–1.48)	0.23	0.79
Design							
Population-based	Overall	9	2,860/4,596	1.21 (1.04–1.40)	1.38 (1.10–1.74)	0.12	0.26
	Caucasian	4	982/2,509	1.26 (0.97–1.63)	1.33 (0.65–2.72)	0.09	0.04
	Asian	2	1,214/2,212	1.42 (0.87–2.31)	1.36 (1.05–1.77)	0.11	0.43
			v				
Hospital-based	Overall	10	4,932/4,762	0.97 (0.83–1.12)	1.17 (1.00–1.36)*	0.09	0.63
	Caucasian	3	4,932/4,762	0.79 (0.60–1.04)	1.15 (0.85–1.55)	0.21	0.54
	Asian	6	2,370/2,378	1.11 (0.94–1.31)	1.23 (1.02–1.48)	0.33	0.72
Histology							
Adenocarcinoma	Overall	8	2,707/4,479	1.14 (0.90–1.45)	1.43 (1.19–1.72)	0.02	0.68
	Caucasian	3	612/2,618	1.05 (0.56–1.97)	1.90 (0.99–3.63)**	0.01	0.30
	Asian	5	2,095/1,861	1.24 (1.04–1.48)	1.38 (1.13–1.69)	0.82	0.79
Squamous cell carcinoma	Overall	6	1,390/3,933	1.02 (0.79–1.33)	1.07 (0.81–1.40)	0.13	0.43
	Caucasian	3	1,008/2,618	1.08 (0.60–1.95)	1.56 (0.54–4.48)	0.03	0.15
	Asian	3	382/1,315	0.96 (0.70–1.31)	1.07 (0.74–1.53)	0.54	0.58
Small cell carcinoma	Asian	2	127/1,194	0.92 (0.55–1.54)	0.99 (0.51–1.93)	0.68	0.52
Overall		19	7,792/9,358	1.07 (0.95–1.21)	1.24 (1.09–1.42)	0.007	0.32

* P = 0.048; ** P = 0.054

## 4. Discussion

Epidemiological studies of common polymorphisms in DNA repair genes, if large and unbiased, can provide insight into the *in vivo* relationships between DNA repair genes and lung cancer risk. Such studies may identify empirical associations which indicate that a polymorphism in a gene of interest has an impact on lung cancer, independent of metabolic regulatory mechanisms and other genetic and environmental variability. Findings from epidemiological studies can complement *in vitro* analyses of the various polymorphisms, genes, and pathways. In addition, epidemiological studies of common polymorphisms can lead to an increased understanding of the public health dimension of DNA-repair variation.

In a narrative review, the *hOGG1* Ser326Cys polymorphism has inconsistently been associated with risk of lung cancer [11]. We conducted a systematic literature review to evaluate the association between the Ser326Cys polymorphism and lung cancer risk. The Cys/Cys genotype was significantly associated with an increased risk of lung cancer risk in Asian populations (OR = 1.27, 95% CI = 1.09–1.48) but not in Caucasian populations (OR = 1.24, 95% CI = 0.84–1.83). There was an increased risk of lung cancer among subjects with the *hOGG1* Cys/Cys genotype, which is consistent with experimental evidence that this isoform exhibits decreased BER activity [40,41]. The meta-analysis of Hung *et al.* showed that the summary OR was 1.37 (95% CI = 1.02–1.82) for the Cys/Cys genotype in various ethnic populations combined (number of studies: 8 [13,14,15,16,17,20,28,29]; number of cases and control: 3,432 and 3,721; ending year of searched publication: 2005; comments in particular: no stratified analysis by ethnicity, inclusion of the studies which were not in the HWE [19,21]) [42]. Like our meta-analysis, the meta-analysis of Li *et al.* also showed that a significantly increased risk was found among Asians carrying the Cys allele (OR = 1.18, 95% CI = 1.01–1.38) (number of studies: 16 [13,14,15,16,17,19,20,21,23,24,28,29,30,40,43,44]; number of cases and control: 6,175 and 6,206; ending year of searched publication: 2007; comments in particular: inclusion of the studies which were not in the HWE [19,21]) [33]. Ethnic difference in the association between lung cancer risk and the *hOGG1* Ser326Cys polymorphism has been suggested. In this study, summary frequencies of the Cys among Caucasians and Asians based on a random effects model were 20.9% (95% CI = 18.9–22.9%) and 46.1% (95% CI = 40.2–52.0%), respectively (Table 2). The frequency of the Cys allele was significantly higher in Asians than in Caucasians (P < 0.001). Based on the previous results of associations between the *hOGG1* Ser326Cys polymorphism and lung cancer, we designated the allele that is presumed to increase the risk of lung cancer as the "at-risk" allele. The ethnic difference in lung cancer risk might be partly due to low prevalence of the Cys allele among Caucasian studies because low frequency of the "at risk" genotype (allele) reduces the statistical power. Another possibility is that this ethnic difference may reflect different gene-environment interactions, gene-gene interactions, or different linkages to the polymorphisms determining lung cancer risk.

In the stratified analysis by histological type of lung cancer, a significant association was found for adenocarcinoma (OR = 1.43, 95% CI = 1.19–1.72) but not for squamous cell carcinoma and small cell carcinoma, although results for small cell carcinoma were inconclusive. Tobacco-specific *N*-nitrosamines (TSNAs), which are formed by *N*-nitrosation of nicotine and other minor alkaloids during tobacco processing and smoking, are major constituents of tobacco smoke and environmental tobacco smoke [45,46]. Some kinds of TSNAs such as 4-(methylnitrosamino)-1-(3-pyridyl)-1-butanone (NNK) induced lung adenocarcinoma in laboratory animals independent of the site of application [47]. NNK induced lung adeno-proliferative lesions through 8-oxoG accumulation and DNA adduct formation [48]. In NNK-treated mice, incidence of lung adenocarcinoma was significantly higher in the *Ogg1* deficient mice than the *Ogg1* positive mice [49]. It is biologically plausible that the low activity genotype of the *hOGG1* Ser326Cys polymorphism (the Cys/Cys genotype) is associated with an increased risk of lung adenocarcinoma. Although hospital patients can provide a relatively economical and convenient source of controls, they might not be representative of the general population. No statistically significant differences of lung cancer risk were identified when stratified by control source although there is a possibility that lung cancer risk may be underestimated in hospital-based case-control studies.

The most important problems facing lung cancer research are identifying "at-risk" individuals and implementing clinical surveillance, prevention practices, and follow-up care. Repair pathways play an important role in lung cancer risk, and genetic variations may contribute to decreased DRC and lung cancer susceptibility. Although the increased/decreased risk associated with individual DNA repair SNPs may be small compared to that conferred by high-penetrance cancer genes, their public health implication may be large because of their high frequency in the general population. It is, thus, essential that epidemiological investigations of DNA repair polymorphisms should be adequately designed. Unfortunately, a fairly good number of studies are limited by their sample size and subsequently suffer from too low power to detect effects that may truly exist. Large and combined analyses may be preferred to minimize the likelihood of both false-positive and false-negative results.

There are some limitations in this meta-analysis. As a meta-analysis pools statistical summaries in the original studies, more reliable estimates can be expected if individual data are available (pooled analysis of raw data). Although we searched several biomedical databases using various search terms, it is possible that relevant citations may have been missed. As well, this study included the focus on English language studies only; there may have been other relevant articles (selection bias). The systematic review, which is limited by the bias against publication of null findings, highlights the complexities inherent in epidemiological research and, particularly, in molecular epidemiological research. The Cys/Cys genotype was associated with about a 30% increase in lung cancer risk. Although the summary risk for developing lung cancer in individuals of each genotype may not be large, lung cancer is such a common malignancy that even a small increase in risk can translate to a large number of excess lung cancer cases. Therefore, polymorphisms, even those not strongly associated with lung cancer, should be considered as potentially important public health issues.

In addition, it is important to keep in mind that a susceptibility factor in one population may not be a factor in another. There are differences in the prevalence of DNA repair polymorphisms across populations. In a population where the prevalence of an "at-risk" genotype in a given polymorphism is very low, the "at-risk" allele or "at-risk" genotype may be too infrequent to assess its associated risk. At a population level, the attributable risk must be small simply because it is an infrequent allele. The major burden of lung cancer in the population probably results from the complex interaction between many genetic and environmental factors over time. The effects of polymorphisms are best represented by their haplotypes. In future association studies on lung cancer, the development of haplotype-based approaches will facilitate the evaluation of haplotypic effects, either for selected polymorphisms physically close to each other or for multiple genes within the same DNA repair pathway. Furthermore, most environmental carcinogens first require metabolic activation by Phase I enzymes to their ultimate forms which then bind to DNA, forming aromatic-DNA adducts that are thought to be an early step in tumorigenesis. On the other hand, these activated forms are detoxified by Phase II enzymes. Thus, genetically determined susceptibility to lung cancer may depend on the metabolic balance among Phase I enzymes, Phase II enzymes and DNA repair enzymes [50]. Further investigations of the combined effects of polymorphisms between DNA repair genes and drug-metabolizing genes may also help to clarify the influence of genetic variation in the carcinogenic process.

## 5. Conclusions

There is evidence that the *hOGG1* Ser326Cys polymorphism play a role in lung carcinogenesis. The Cys/Cys genotype was associated with about a 30% increase in lung cancer risk. Although the summary risk for developing lung cancer in individuals of the genotype may not be large, lung cancer is such a common malignancy that even a small increase in risk can translate to a large number of excess lung cancer cases. Therefore, a polymorphism—even one not strongly associated with lung cancer—should be considered as a potentially important public health issue. As lung cancer is a multifactorial disease, further investigations of the gene-gene and gene-environment interactions on the *hOGG1* polymorphism-associated lung cancer risk may help to better understand the molecular pathogenesis of human lung cancer.
